# Supplementary material for: Iterative improvement in the automatic modular design of robot swarms
Source: PeerJ Comput Sci. 2020 Dec 7;6:e322. doi: 10.7717/peerj-cs.322 (PMC7924708; doi:10.7717/peerj-cs.322)
Supplement: Supplemental Information 3 [file peerj-cs-06-322-s003.zip › argos3/doc/api/standalone/a00418_source.html]

ARGoS: plugins/robots/e-puck/simulator/qtopengl\_epuck.cpp Source File


- Main Page
- Related Pages
- Namespaces
- Classes
- Files

- File List
- File Members

# plugins/robots/e-puck/simulator/qtopengl\_epuck.cpp

Go to the documentation of this file.

```
00001 
00007 #include "qtopengl_epuck.h"
00008 #include "epuck_entity.h"
00009 #include <argos3/core/simulator/entity/embodied_entity.h>
00010 #include <argos3/core/utility/math/vector2.h>
00011 #include <argos3/core/utility/math/vector3.h>
00012 #include <argos3/plugins/simulator/entities/led_equipped_entity.h>
00013 #include <argos3/plugins/simulator/visualizations/qt-opengl/qtopengl_widget.h>
00014 
00015 namespace argos {
00016 
00017    /****************************************/
00018    /****************************************/
00019 
00020    /* All measures are in meters */
00021 
00022    static const Real WHEEL_DIAMETER              = 0.041f;
00023    static const Real WHEEL_RADIUS                = WHEEL_DIAMETER * 0.5f;
00024    static const Real WHEEL_WIDTH                 = 0.01f;
00025    static const Real HALF_WHEEL_WIDTH            = WHEEL_WIDTH * 0.5f;
00026    static const Real INTERWHEEL_DISTANCE         = 0.053f;
00027    static const Real HALF_INTERWHEEL_DISTANCE    = INTERWHEEL_DISTANCE * 0.5f;
00028 
00029    static const Real CHASSIS_ELEVATION           = 0.005f;                            // to be checked!
00030    static const Real HALF_CHASSIS_LENGTH         = 0.0275f;                             // to be checked!
00031    static const Real HALF_CHASSIS_WIDTH          = HALF_INTERWHEEL_DISTANCE - HALF_WHEEL_WIDTH;
00032 
00033    static const Real BODY_RADIUS                 = 0.035f;
00034    static const Real BODY_ELEVATION              = WHEEL_DIAMETER + CHASSIS_ELEVATION; // to be checked!
00035    static const Real BODY_HEIGHT                 = 0.03f;                              // to be checked!
00036 
00037    static const Real LED_ELEVATION               = BODY_ELEVATION + BODY_HEIGHT;
00038    static const Real LED_HEIGHT                  = 0.01;                               // to be checked!
00039    static const Real LED_UPPER_RING_INNER_RADIUS = 0.8 * BODY_RADIUS;
00040 
00041    /****************************************/
00042    /****************************************/
00043 
00044    CQTOpenGLEPuck::CQTOpenGLEPuck() :
00045       m_unVertices(40),
00046       m_fLEDAngleSlice(360.0f / 8.0f) {
00047       /* Reserve the needed display lists */
00048       m_unLists = glGenLists(4);
00049 
00050       /* Assign indices for better referencing (later) */
00051       m_unWheelList   = m_unLists;
00052       m_unChassisList = m_unLists + 1;
00053       m_unBodyList    = m_unLists + 2;
00054       m_unLEDList     = m_unLists + 3;
00055 
00056       /* Create the wheel display list */
00057       glNewList(m_unWheelList, GL_COMPILE);
00058       RenderWheel();
00059       glEndList();
00060 
00061       /* Create the body display list */
00062       glNewList(m_unBodyList, GL_COMPILE);
00063       RenderBody();
00064       glEndList();
00065 
00066       /* Create the chassis display list */
00067       glNewList(m_unChassisList, GL_COMPILE);
00068       RenderChassis();
00069       glEndList();
00070 
00071       /* Create the LED display list */
00072       glNewList(m_unLEDList, GL_COMPILE);
00073       RenderLED();
00074       glEndList();
00075    }
00076 
00077    /****************************************/
00078    /****************************************/
00079 
00080    CQTOpenGLEPuck::~CQTOpenGLEPuck() {
00081       glDeleteLists(m_unLists, 4);
00082    }
00083 
00084    /****************************************/
00085    /****************************************/
00086 
00087    void CQTOpenGLEPuck::Draw(CEPuckEntity& c_entity) {
00088       /* Place the chassis */
00089       glCallList(m_unChassisList);
00090       /* Place the body */
00091       glCallList(m_unBodyList);
00092       /* Place the wheels */
00093       glPushMatrix();
00094       glTranslatef(0.0f, HALF_INTERWHEEL_DISTANCE, 0.0f);
00095       glCallList(m_unWheelList);
00096       glPopMatrix();
00097       glPushMatrix();
00098       glTranslatef(0.0f, -HALF_INTERWHEEL_DISTANCE, 0.0f);
00099       glCallList(m_unWheelList);
00100       glPopMatrix();
00101       /* Place the LEDs */
00102       glPushMatrix();
00103       CLEDEquippedEntity& cLEDEquippedEntity = c_entity.GetLEDEquippedEntity();
00104       for(UInt32 i = 0; i < 8; i++) {
00105          const CColor& cColor = cLEDEquippedEntity.GetLED(i).GetColor();
00106          glRotatef(-m_fLEDAngleSlice, 0.0f, 0.0f, 1.0f);
00107          SetLEDMaterial(cColor.GetRed(),
00108                         cColor.GetGreen(),
00109                         cColor.GetBlue());
00110          glCallList(m_unLEDList);
00111       }
00112       glPopMatrix();
00113    }
00114 
00115    /****************************************/
00116    /****************************************/
00117 
00118    void CQTOpenGLEPuck::SetGreenPlasticMaterial() {
00119       const GLfloat pfColor[]     = {   0.0f, 1.0f, 0.0f, 1.0f };
00120       const GLfloat pfSpecular[]  = {   0.9f, 0.9f, 0.9f, 1.0f };
00121       const GLfloat pfShininess[] = { 100.0f                   };
00122       const GLfloat pfEmission[]  = {   0.0f, 0.0f, 0.0f, 1.0f };
00123       glMaterialfv(GL_FRONT_AND_BACK, GL_AMBIENT_AND_DIFFUSE, pfColor);
00124       glMaterialfv(GL_FRONT_AND_BACK, GL_SPECULAR,            pfSpecular);
00125       glMaterialfv(GL_FRONT_AND_BACK, GL_SHININESS,           pfShininess);
00126       glMaterialfv(GL_FRONT_AND_BACK, GL_EMISSION,            pfEmission);
00127    }
00128 
00129    /****************************************/
00130    /****************************************/
00131 
00132    void CQTOpenGLEPuck::SetRedPlasticMaterial() {
00133       const GLfloat pfColor[]     = {   1.0f, 0.0f, 0.0f, 1.0f };
00134       const GLfloat pfSpecular[]  = {   0.9f, 0.9f, 0.9f, 1.0f };
00135       const GLfloat pfShininess[] = { 100.0f                   };
00136       const GLfloat pfEmission[]  = {   0.0f, 0.0f, 0.0f, 1.0f };
00137       glMaterialfv(GL_FRONT_AND_BACK, GL_AMBIENT_AND_DIFFUSE, pfColor);
00138       glMaterialfv(GL_FRONT_AND_BACK, GL_SPECULAR,            pfSpecular);
00139       glMaterialfv(GL_FRONT_AND_BACK, GL_SHININESS,           pfShininess);
00140       glMaterialfv(GL_FRONT_AND_BACK, GL_EMISSION,            pfEmission);
00141    }
00142 
00143    /****************************************/
00144    /****************************************/
00145 
00146    void CQTOpenGLEPuck::SetCircuitBoardMaterial() {
00147       const GLfloat pfColor[]     = { 0.0f, 0.0f, 1.0f, 1.0f };
00148       const GLfloat pfSpecular[]  = { 0.5f, 0.5f, 1.0f, 1.0f };
00149       const GLfloat pfShininess[] = { 10.0f                  };
00150       const GLfloat pfEmission[]  = { 0.0f, 0.0f, 0.0f, 1.0f };
00151       glMaterialfv(GL_FRONT_AND_BACK, GL_AMBIENT_AND_DIFFUSE, pfColor);
00152       glMaterialfv(GL_FRONT_AND_BACK, GL_SPECULAR,            pfSpecular);
00153       glMaterialfv(GL_FRONT_AND_BACK, GL_SHININESS,           pfShininess);
00154       glMaterialfv(GL_FRONT_AND_BACK, GL_EMISSION,            pfEmission);
00155    }
00156 
00157    /****************************************/
00158    /****************************************/
00159 
00160    void CQTOpenGLEPuck::SetLEDMaterial(GLfloat f_red,
00161                                        GLfloat f_green,
00162                                        GLfloat f_blue) {
00163       const GLfloat fEmissionFactor = 10.0f;
00164       const GLfloat pfColor[]     = {                    f_red,                   f_green,                   f_blue, 1.0f };
00165       const GLfloat pfSpecular[]  = {                     0.0f,                      0.0f,                     0.0f, 1.0f };
00166       const GLfloat pfShininess[] = {                     0.0f                                                            };
00167       const GLfloat pfEmission[]  = {  f_red * fEmissionFactor, f_green * fEmissionFactor, f_blue * fEmissionFactor, 1.0f };
00168       glMaterialfv(GL_FRONT_AND_BACK, GL_AMBIENT_AND_DIFFUSE, pfColor);
00169       glMaterialfv(GL_FRONT_AND_BACK, GL_SPECULAR,            pfSpecular);
00170       glMaterialfv(GL_FRONT_AND_BACK, GL_SHININESS,           pfShininess);
00171       glMaterialfv(GL_FRONT_AND_BACK, GL_EMISSION,            pfEmission);
00172    }
00173 
00174    /****************************************/
00175    /****************************************/
00176 
00177    void CQTOpenGLEPuck::RenderWheel() {
00178       /* Set material */
00179       SetRedPlasticMaterial();
00180       /* Right side */
00181       CVector2 cVertex(WHEEL_RADIUS, 0.0f);
00182       CRadians cAngle(CRadians::TWO_PI / m_unVertices);
00183       CVector3 cNormal(-1.0f, -1.0f, 0.0f);
00184       cNormal.Normalize();
00185       glBegin(GL_POLYGON);
00186       for(GLuint i = 0; i <= m_unVertices; i++) {
00187          glNormal3f(cNormal.GetX(), cNormal.GetY(), cNormal.GetZ());
00188          glVertex3f(cVertex.GetX(), -HALF_WHEEL_WIDTH, WHEEL_RADIUS + cVertex.GetY());
00189          cVertex.Rotate(cAngle);
00190          cNormal.RotateY(cAngle);
00191       }
00192       glEnd();
00193       /* Left side */
00194       cVertex.Set(WHEEL_RADIUS, 0.0f);
00195       cNormal.Set(-1.0f, 1.0f, 0.0f);
00196       cNormal.Normalize();
00197       cAngle = -cAngle;
00198       glBegin(GL_POLYGON);
00199       for(GLuint i = 0; i <= m_unVertices; i++) {
00200          glNormal3f(cNormal.GetX(), cNormal.GetY(), cNormal.GetZ());
00201          glVertex3f(cVertex.GetX(), HALF_WHEEL_WIDTH, WHEEL_RADIUS + cVertex.GetY());
00202          cVertex.Rotate(cAngle);
00203          cNormal.RotateY(cAngle);
00204       }
00205       glEnd();
00206       /* Tire */
00207       cNormal.Set(1.0f, 0.0f, 0.0f);
00208       cVertex.Set(WHEEL_RADIUS, 0.0f);
00209       cAngle = -cAngle;
00210       glBegin(GL_QUAD_STRIP);
00211       for(GLuint i = 0; i <= m_unVertices; i++) {
00212          glNormal3f(cNormal.GetX(), cNormal.GetY(), cNormal.GetZ());
00213          glVertex3f(cVertex.GetX(), -HALF_WHEEL_WIDTH, WHEEL_RADIUS + cVertex.GetY());
00214          glVertex3f(cVertex.GetX(),  HALF_WHEEL_WIDTH, WHEEL_RADIUS + cVertex.GetY());
00215          cVertex.Rotate(cAngle);
00216          cNormal.RotateY(cAngle);
00217       }
00218       glEnd();
00219    }
00220 
00221    /****************************************/
00222    /****************************************/
00223 
00224    void CQTOpenGLEPuck::RenderChassis() {
00225       /* Set material */
00226       SetGreenPlasticMaterial();
00227       /* This part covers the bottom face (parallel to XY) */
00228       glBegin(GL_QUADS);
00229       /* Bottom face */
00230       glNormal3f(0.0f, 0.0f, -1.0f);
00231       glVertex3f( HALF_CHASSIS_LENGTH,  HALF_CHASSIS_WIDTH, CHASSIS_ELEVATION);
00232       glVertex3f( HALF_CHASSIS_LENGTH, -HALF_CHASSIS_WIDTH, CHASSIS_ELEVATION);
00233       glVertex3f(-HALF_CHASSIS_LENGTH, -HALF_CHASSIS_WIDTH, CHASSIS_ELEVATION);
00234       glVertex3f(-HALF_CHASSIS_LENGTH,  HALF_CHASSIS_WIDTH, CHASSIS_ELEVATION);
00235       glEnd();
00236       /* This part covers the faces (South, East, North, West) */
00237       glBegin(GL_QUAD_STRIP);
00238       /* Starting side */
00239       glNormal3f(-1.0f, 0.0f, 0.0f);
00240       glVertex3f(-HALF_CHASSIS_LENGTH, -HALF_CHASSIS_WIDTH, CHASSIS_ELEVATION + WHEEL_DIAMETER);
00241       glVertex3f(-HALF_CHASSIS_LENGTH, -HALF_CHASSIS_WIDTH, CHASSIS_ELEVATION);
00242       /* South face */
00243       glVertex3f( HALF_CHASSIS_LENGTH, -HALF_CHASSIS_WIDTH, CHASSIS_ELEVATION + WHEEL_DIAMETER);
00244       glVertex3f( HALF_CHASSIS_LENGTH, -HALF_CHASSIS_WIDTH, CHASSIS_ELEVATION);
00245       /* East face */
00246       glNormal3f(0.0f, -1.0f, 0.0f);
00247       glVertex3f( HALF_CHASSIS_LENGTH,  HALF_CHASSIS_WIDTH, CHASSIS_ELEVATION + WHEEL_DIAMETER);
00248       glVertex3f( HALF_CHASSIS_LENGTH,  HALF_CHASSIS_WIDTH, CHASSIS_ELEVATION);
00249       /* North face */
00250       glNormal3f(1.0f, 0.0f, 0.0f);
00251       glVertex3f(-HALF_CHASSIS_LENGTH,  HALF_CHASSIS_WIDTH, CHASSIS_ELEVATION + WHEEL_DIAMETER);
00252       glVertex3f(-HALF_CHASSIS_LENGTH,  HALF_CHASSIS_WIDTH, CHASSIS_ELEVATION);
00253       /* West face */
00254       glNormal3f(0.0f, 1.0f, 0.0f);
00255       glVertex3f(-HALF_CHASSIS_LENGTH, -HALF_CHASSIS_WIDTH, CHASSIS_ELEVATION + WHEEL_DIAMETER);
00256       glVertex3f(-HALF_CHASSIS_LENGTH, -HALF_CHASSIS_WIDTH, CHASSIS_ELEVATION);
00257       glEnd();
00258    }
00259 
00260    /****************************************/
00261    /****************************************/
00262 
00263    void CQTOpenGLEPuck::RenderBody() {
00264       /* Set material */
00265       SetGreenPlasticMaterial();
00266       CVector2 cVertex(BODY_RADIUS, 0.0f);
00267       CRadians cAngle(-CRadians::TWO_PI / m_unVertices);
00268       /* Bottom part */
00269       glBegin(GL_POLYGON);
00270       glNormal3f(0.0f, 0.0f, -1.0f);
00271       for(GLuint i = 0; i <= m_unVertices; i++) {
00272          glVertex3f(cVertex.GetX(), cVertex.GetY(), BODY_ELEVATION);
00273          cVertex.Rotate(cAngle);
00274       }
00275       glEnd();
00276       /* Side surface */
00277       cAngle = -cAngle;
00278       CVector2 cNormal(1.0f, 0.0f);
00279       cVertex.Set(BODY_RADIUS, 0.0f);
00280       glBegin(GL_QUAD_STRIP);
00281       for(GLuint i = 0; i <= m_unVertices; i++) {
00282          glNormal3f(cNormal.GetX(), cNormal.GetY(), 0.0f);
00283          glVertex3f(cVertex.GetX(), cVertex.GetY(), BODY_ELEVATION + BODY_HEIGHT);
00284          glVertex3f(cVertex.GetX(), cVertex.GetY(), BODY_ELEVATION);
00285          cVertex.Rotate(cAngle);
00286          cNormal.Rotate(cAngle);
00287       }
00288       glEnd();
00289       /* Top part */
00290       glBegin(GL_POLYGON);
00291       cVertex.Set(LED_UPPER_RING_INNER_RADIUS, 0.0f);
00292       glNormal3f(0.0f, 0.0f, 1.0f);
00293       for(GLuint i = 0; i <= m_unVertices; i++) {
00294          glVertex3f(cVertex.GetX(), cVertex.GetY(), BODY_ELEVATION + BODY_HEIGHT + LED_HEIGHT);
00295          cVertex.Rotate(cAngle);
00296       }
00297       glEnd();
00298       /* Triangle to set the direction */
00299       SetLEDMaterial(1.0f, 1.0f, 0.0f);
00300       glBegin(GL_TRIANGLES);
00301       glVertex3f( BODY_RADIUS * 0.7,               0.0f, BODY_ELEVATION + BODY_HEIGHT + LED_HEIGHT + 0.001f);
00302       glVertex3f(-BODY_RADIUS * 0.7,  BODY_RADIUS * 0.3, BODY_ELEVATION + BODY_HEIGHT + LED_HEIGHT + 0.001f);
00303       glVertex3f(-BODY_RADIUS * 0.7, -BODY_RADIUS * 0.3, BODY_ELEVATION + BODY_HEIGHT + LED_HEIGHT + 0.001f);
00304       glEnd();
00305    }
00306 
00307    /****************************************/
00308    /****************************************/
00309 
00310    void CQTOpenGLEPuck::RenderLED() {
00311       /* Side surface */
00312       CVector2 cVertex(BODY_RADIUS, 0.0f);
00313       CRadians cAngle(CRadians::TWO_PI / m_unVertices);
00314       CVector2 cNormal(1.0f, 0.0f);
00315       glBegin(GL_QUAD_STRIP);
00316       for(GLuint i = 0; i <= m_unVertices / 8; i++) {
00317          glNormal3f(cNormal.GetX(), cNormal.GetY(), 0.0f);
00318          glVertex3f(cVertex.GetX(), cVertex.GetY(), LED_ELEVATION + LED_HEIGHT);
00319          glVertex3f(cVertex.GetX(), cVertex.GetY(), LED_ELEVATION);
00320          cVertex.Rotate(cAngle);
00321          cNormal.Rotate(cAngle);
00322       }
00323       glEnd();
00324       /* Top surface  */
00325       cVertex.Set(BODY_RADIUS, 0.0f);
00326       CVector2 cVertex2(LED_UPPER_RING_INNER_RADIUS, 0.0f);
00327       glBegin(GL_QUAD_STRIP);
00328       glNormal3f(0.0f, 0.0f, 1.0f);      
00329       for(GLuint i = 0; i <= m_unVertices / 8; i++) {         
00330          glVertex3f(cVertex2.GetX(), cVertex2.GetY(), BODY_ELEVATION + BODY_HEIGHT + LED_HEIGHT);
00331          glVertex3f(cVertex.GetX(), cVertex.GetY(), BODY_ELEVATION + BODY_HEIGHT + LED_HEIGHT);
00332          cVertex.Rotate(cAngle);
00333          cVertex2.Rotate(cAngle);
00334       }
00335       glEnd();
00336    }
00337 
00338    /****************************************/
00339    /****************************************/
00340 
00341    class CQTOpenGLOperationDrawEPuckNormal : public CQTOpenGLOperationDrawNormal {
00342    public:
00343       void ApplyTo(CQTOpenGLWidget& c_visualization,
00344                    CEPuckEntity& c_entity) {
00345          static CQTOpenGLEPuck m_cModel;
00346          c_visualization.DrawRays(c_entity.GetControllableEntity());
00347          c_visualization.DrawEntity(c_entity.GetEmbodiedEntity());
00348          m_cModel.Draw(c_entity);
00349       }
00350    };
00351 
00352    class CQTOpenGLOperationDrawEPuckSelected : public CQTOpenGLOperationDrawSelected {
00353    public:
00354       void ApplyTo(CQTOpenGLWidget& c_visualization,
00355                    CEPuckEntity& c_entity) {
00356          c_visualization.DrawBoundingBox(c_entity.GetEmbodiedEntity());
00357       }
00358    };
00359 
00360    REGISTER_QTOPENGL_ENTITY_OPERATION(CQTOpenGLOperationDrawNormal, CQTOpenGLOperationDrawEPuckNormal, CEPuckEntity);
00361 
00362    REGISTER_QTOPENGL_ENTITY_OPERATION(CQTOpenGLOperationDrawSelected, CQTOpenGLOperationDrawEPuckSelected, CEPuckEntity);
00363 
00364    /****************************************/
00365    /****************************************/
00366 
00367 }
```

---

Generated on 10 Jul 2018 for ARGoS by 
 1.6.1
